# Factors Associated with Evidence-Based Decision-Making Among Specialized Nurses Working in Selected Health Facilities in Nairobi, Kenya

**DOI:** 10.24248/eahrj.v9i1.839

**Published:** 2025-09-30

**Authors:** Safari Agure, Wanja Tenamburgen, Lillian Muiruri, Erastus Muniu

**Affiliations:** a Kenya Methodist University, Nairobi, Kenya; b Kenya Medical Research Institute, Nairobi, Kenya.

## Abstract

**Background:**

Evidence-Based Decision-Making (EBDM) is central to quality nursing practice, yet many nurses continue to rely on intuition when making clinical decisions. In low-resource settings such as Kenya, limited access to information, knowledge-sharing barriers, and institutional constraints further hinder EBDM. Understanding the factors that influence EBDM among specialized nurses is vital for improving patient care and health system outcomes.

**Methods:**

This study employed a concurrent nested mixed-methods design guided by the ACE Star Model. The quantitative arm utilized a cross-sectional census of 51 maternal and child health nurses from four public hospitals in Nairobi, while the qualitative arm involved two in-depth interviews with supervisors. Data were collected using a structured questionnaire and interview guide. Quantitative analysis was conducted using SPSS with logistic regression to identify factors associated with EBDM, while qualitative data were analyzed thematically.

**Results:**

The overall response rate was 100%. Most respondents (72.5% female) were diploma holders with a median age of 30 years. Findings revealed that the predominant reliance on intuition was significantly associated with low utilization of EBDM (*p*=.039). Individual factors, including skills, incentives, and research literacy, were strongly associated with EBDM utilization (*p*=.001). Married nurses and those with higher academic qualifications demonstrated marginally lower odds of low EBDM utilisation. Institutional factors and barriers such as limited internet access, staffing constraints, and time shortages were reported but not statistically significant. Qualitative insights highlighted reliance on tacit knowledge, bounded autonomy, and workload pressures as major influences on decision-making.

**Conclusion:**

Specialized nurses in Nairobi largely rely on tacit knowledge and intuition, with individual capacities playing a critical role in shaping EBDM. Strengthening research skills, integrating EBDM training early into nursing curricula, and creating institutional support systems are essential for enhancing evidence use in clinical practice. Strategies for capturing and translating tacit knowledge into actionable evidence should be prioritized to improve patient outcomes. Further large-scale, multidisciplinary studies are recommended to validate these findings.

## BACKGROUND

Making a choice from a range of options is at the core of decision-making and Evidence-Based Decision-making (EBDM). Working within larger multidisciplinary teams, nurses are responsible for making judgments and decisions to determine clinical outcomes for patients. Their decisions are complex due in part to the diversity of patients, technological advances, and practice settings.^1^ This makes EBDM and the use of knowledge that is based on science to guide them in making correct decisions and problem-solving critical.^2^

To strengthen decision-making, professionalism, expertise building, and service delivery, access to information is vital. The aim of collecting data through research is to ensure the effective and appropriate use of resources to facilitate health interventions. Research outcomes include data on population health and service provision for use in decision-making and planning for the health system, especially from where data was collected. However, this may not always be the case.

The focus of research utilisation is shifting from traditional knowledge dissemination which is linear (science push model) to knowledge translation (KT). This is in recognition of the importance of applying research to decision making and eventually implementation, however, the traditional, approach still remains the most commonly used.^3^ The Knowledge to Action (K2A) framework has been proven as a useful planning, reflecting and evaluation tool to assess ways in which healthcare outcomes can be improved through KT.^4^ By readily providing access to health information, KT can facilitate effective health services’ provision and products that can strengthen the health care system. Evidence based practice (EBP), an off shoot of EBDM and the basis for EBDM&P (Evidence Based Decision Making & Practice) an acronym that may be referred to synonymously henceforth, specifically educates nursing professionals in how to find the research information that they need for developing, implementing, and evaluating proven programs or policies for positive health outcomes.

It is however not clear what sources of information nurses use to guide their clinical decision-making.^8^ New information that comes from research can approve/critique existing information, beliefs, and values for nurses who are noted as the largest health professionals in the workforce^9^ and reduce the uncertainty in the sector. In low-resource settings there is low utilization of local data for planning in a health system, monitoring, evaluation and decision-making, this being a running challenge. This is in part because of limited information and knowledge sharing, inadequate staff capacity to analyze and use data in decision-making and non-collaborative decision making.^5, 6, 7, 8^

The history of engagement of nurses with evidence in nursing practice dates back to Florence Nightingale in the 1800s and has since evolved.^10^ This engagement with research requires that nurses be more proactive in accessing, appraising and incorporating research into their decision making so that the gap in theory and practice can continue to close and enable the nursing profession to advance.^10^ The primary motive for nurses to engage with research-based evidence is to reduce uncertainty in their decision making and practice. The concept of shared decision making (SDM) provides platforms for nurses to support basic needs (care and information) of their patients and assist in steering key decisions.^11^

Roles that nurses have been seen to play in decision-making for patient care include being a part of an inter-professional team for provision of care^12^, decision coaching, advocating for patient's preferences and uncertainty in decision making among nurses exists; more so because judgments are characterised by complexity and unpredictability. Nurses make judgments & decisions that have direct or indirect impact to patient care and outcomes sometimes impacting fatalities.

### Research Gap

In Kenya, research conducted with nurses as respondents has concentrated on enhancing the implementation arena of clinical practice; hence, little evidence has been unearthed about the process of decision-making among nurses. Anecdotal information, however, alludes to the application of intuition-based information rather than evidence-based information as majorly guiding practice in nurses’ decision-making and service delivery. However, best practice advocates that clinical decision making be more Evidence based, through KT. This results in a gap of research in KT within the specialized nursing field that needs to be closed for the improvement and efficiency of decision making and henceforth improved service delivery in these specialized areas.

### Objective

To determine the factors related to evidence-based decision-making (EBDM) & practice among maternal and child health nurses working in selected health facilities in Nairobi, Kenya.

### Variable

Various factors have been associated with EBDM. The study selected four of these factors to act as independent variables. These factors were individual factors, institutional factors, thought paradigms (Intuition vs evidence) and barriers to access of evidence. Indicators for the factors were intuition versus evidence thought paradigms, Individual factors, institutional factors and barriers to access of evidence.

## METHODOLOGY

### Research Design

The study took a concurrent nested mixed methods design that enabled the complex phenomenon of decision-making be studied in an in-depth, multilayered and multipart manner. The quantitative phase was the primary and dormant phase, while the qualitative phase was embedded within.^13^ Quantitative phase was cross-sectional in nature with qualitative utilizing grounded theory applying the ACE star theory to conceptualize the study factors and variables. As a framework, the ACE star provides pathways for turning complex research into structured, actionable steps required for evidence-based decision-making to turn knowledge into action.

### Study Site and Population

The study was carried out in Nairobi County, the capital city of Kenya, in Kenyatta National Hospital, Mbagathi Hospital, Pumwani Hospital and Mama Lucy Kibaki Hospital. The study had a target population of maternal and child health nurses working in reproductive wards in the selected facilities as well as their supervisors.

### Sampling

Parallel sampling^14^ was used. However since the currently registered reproductive nurses working in the selected facilities are less than 1000 in population, for the four facilities, a census was therefore carried out for the quantitative data where all nurses available in the facility during the study site visit were invited to take part and recruited. For the qualitative phase of the study, 2 key informants’ interviews were conducted from 2 facilities. This was adequate to reach saturation point. This sample was purposively selected. Recruitment was done according to the sampling frame in Table 1.

**TABLE 1:** Sampling Frame

Facility	Target Population	Sample Achieved
Kenyatta National Hospital	All available	24
Mbagathi District Hospital	All available	11
Pumwani Maternity Hospital	All available	6
Mama Lucy Kibaki Hospital	All available	10
TOTAL		51

### Data Collection and Analysis

Data collection was done between the dates of 19/03/2024 and 06/5/2024. A survey questionnaire was developed by the principal researcher through a multi-step process. This process included literature review, stakeholder consultation, and pre-testing. Relevant variables were identified through the ACE star framework and indicators through literature aligned to the study objective. Cultural relevance was determined through a pre-test with a small sample from a similar level 4 facility in Kiambu county to assess question flow, timing, and response clarity. Feedback from the pre-test informed final adjustments to the tool before full deployment.

To test for internal consistency of the quantitative tool, Cronbach alpha was used. A figure of α < 0.7 was considered acceptable. SPSS was used for quantitative Univariate, Bivariate and Multivariate analysis while Qual interviews were transcribed and translated for manual analysis thematically.

Exploratory data analysis (EDA) was employed at the initial stage of analysis to identify the normal distribution of variables, missing data, and extreme outliers for quantitative data.

Qualitative interviews were held at the convenience of the respondents within the facilities. At least one interview was targeted for each of the 4 facilities or until saturation is reached. However saturation point was reached after the second interview as the information being given was similar. Interviews were audio recorded with permission of respondent for later transcription, ethical considerations being adhered to through the informed consent form. No names were used during the interviews with participant numbers used to maintain confidentiality.

### Ethical Considerations

The study was presented to and approved by the research review board of Kenya Methodist university (KEMU) prior to being conducted to ensure that the elements conform to the principles of good practice of research. This study was given an ethics clearance with number KeMU/ISERC/HSM/27/2023. Any other required permission or permit including from the National Commission for Science, Technology and Innovation (NACOSTI) and institutional permissions, were acquired through the NACOSTI permit reference number NACOSTI/P/23/3294. No invasive procedures were used, hence minimal risk was anticipated. All information given was kept confidential by the Principle Investigator. All the participants signed an informed consent form to ensure voluntariness in the study, not coercion. As the survey respondents were adults, they signed 2 copies of the consent; one copy for the researcher the other for themselves, prior to completing the questionnaire. For the qualitative research, additional oral consent was given due to the fact that audio recording was done. For interviews, audio recording was done with ethical considerations adhered to through the same informed consent form. No names were recorded during the interviews, but instead participants were assigned numbers in order to maintain confidentiality. Within this consent form, all details of the study including investigators and their contacts as well as study procedures were given.

## RESULTS

### Quantitative Results

A likert scale instrument was designed by the researcher based on the factors adapted from the ACE model and used to identify factors to be adapted into the study depending on context. The factors adapted are Cronbach alpha internal consistency for the study tool was 0.8774. An alpha measure of α 0.8 ≤ α < 0.9 was pre-determined as acceptable. As ≤ 0.8.774 was realized, the Cronchbach tests determined the instrument as valid. All the nurses invited to the study agreed to and completed the questionnaires. As the study quantitative aspects applied a census, hence the response rate can be reported as 100%.

### Quantitative Analysis

Analysis of social demographics was done through Univariate analysis. With a statistical significance set at p < .05, all p-values less than this threshold were considered statistically significant. The aspects analyzed are the mean age of respondents, average salary and educational levels. A total of 51 nurses (72.5% females and 27.5% males) participated in the study. Their age ranged from 25 to 58 years with a median of 30 years and an interquartile range (IQR) of 28 to 38 years. The majority, 64.7%, were married, and slightly over three quarters (76.5%) were diploma holders and below. The scores for the independent variables are given in Table 2.

**TABLE 2:** Distribution of Scores for the Independent Factors

Factor	N	Summed Scores Median	Lower IQR^a^	Upper IQR	Minimum	Maximum
Evidence vs intuition	51	13	11	15	7	23
Institutional factors	51	24	20.5	29.5	9	38
Individual factors	51	13	9.5	16	6	25
Barriers to access	51	16	14	19	5	22

a IQR, Interquartile range.

Socio demographic and individual factors associated with nurses’ low level EBDM utilization were analyzed using binary logistic regression to identify low level EBDM users correlates. Low level EBDM utilization was found to be independently associated with marital status, education level and individual factors as they were retained in the final model. The odds of having low level EBDM utilization was lower 0.226 for married compared to not married though marginally significant (*p*=.065). The Basic degree holders had 5.69 higher odds of having low level EBDM utilization compared to Diploma holders & below though marginally significant (.069). The odds ratio for individual factors (continuous variable), indicates that every unit increase in individual factor score is associated with a 32.5% increase in the odds of having low level EBDM utilization which is statistically significant (*p*=.003). Tables 3 & 4.

**TABLE 3:** Factors Associated With Nurses’ Low Level EBDM Utilisation (N=51)

Characteristic/Factor	EBDM Utilisation		*P Value*	Parameter Estimates		Upper	*P Value*
	High level	Low level		AOR^a^	95% Cl^b^		
	n=23%	n=28%		Lower			
Sex							
Male	21.7	32.1	.407				
Female	78.3	67.9					
Marital status							
Not married^c^	17.4	50.0	.015	1			
Married	82.6	50.0		0.226	0.047	1.095	.065
Education level							
Diploma holder & below^c^	87.0	67.9	.110	1			
Basic degree holder	13.0	32.1	5.693	0.872	37.178		.069
Employment duration							
Less than 5 years	60.9	60.7	.991				
6 years and above	39.1	39.3					
Monthly income							
Kes 50,000 & below	30.4	25.0	.863				
Between Kes 51,000 & Kes 80,000	43.5	42.9					
Above Kes 80,000	26.1	32.1					
Age and factor scores	Median (IQR)^d^	Median (IQR)^d^					
Age last birthday in years	30 (29–35)	30 (28–41)	.731				
Evidence vs intuition	12 (10–14.5)	14 (12–17)	.039	1.086	0.873	1.353	.429
Institutional	22 (18.5–28)	26.5 (21.5–29.5)	.263				
Individual	10 (8–13.5)	16 (12.5–18)	<.001	1.325	1.103	1.592	.003
Barriers to access	14 (12–18)	17.5 (14.5–19.5)	.146	0.889	0.729	1.083	.243

a AOR, Adjusted Odds Ratio;

b 95% CI, 95% Confidence Interval;

c Reference category;

d IQR, Interquartile range.

**TABLE 4:** Responses to EBDM Utilization Items or Dependent Variable (N=51)

Item^*^	n	Responses				
		Strongly Agree, %	Slightly Agree, %	Neutral, %	Slightly Disagree, %	Strongly Disagree, %
3.1	51	76.5	5.9	9.8	2.0	5.9
3.2	51	60.8	19.6	19.6	0	0
3.3	51	82.4	5.9	9.8	0	2.0
3.4	51	45.1	19.6	25.5	7.8	2.0
3.5	51	68.6	11.8	17.6	2.0	0
3.6	51	58.8	9.8	25.5	3.9	2.0

* Items for EBDM utilisation:
3.1 - The care that I give have an impact on patient outcomes 3.2 - The care I give my patients are based on evidence 3.3 - I feel satisfied with my job when my patients have positive outcomes from my care 3.4 - The support for decision making I get from the use of evidence reduces fatigue and burnout 3.5 - I am confident in my care abilities when my decisions and practice are evidence based. 3.6 - I am confident of my knowledge in dealing with patient situation

### Qualitative Analysis

Qualitative data were collected through 2 in-depth interviews with identified nurses’ supervisors at each health facility. were conducted. The interviews were audio recorded and then transcribed verbatim and analyzed using thematic analysis, a method for identifying, analyzing, and reporting patterns (themes) within data. This approach was chosen for its flexibility and its ability to provide rich, detailed, and complex accounts of participant perspectives. Full results of these interviews are given in TABLE 5.

**TABLE 5:** Qualitative Results

Theme	Sub Theme		Representative quotes	
			Respondent 1	Respondent 2
Evidence-Based Practice & Decision-Making	**Knowledge**	There is emphasis on the importance of using evidence-based practices in nursing…	“… Yes something showing that this is what is supposed to be done. …. Those are evidence-based & have been proven that they work?”.	“According to my understanding this concept is about using evidence in making decisions to improve patients care. These are normally used in work improvement teams”.
	**Examples of EBDM tools**	They discuss the use of protocols, guidelines, & tools developed from evidence-based research to improve patient care	“Evidence-based we use SOPs… We use … protocols, we use comprehensive neonatal booklets to which we refer to if it is a given condition you want to manage; how are we doing”	“…through the automated tools which in our case is a kathograph and be able to intervene and act” “We have come up with simplified tools for PPH managing PPH which are easier to follow than the IEC materials which are able to guide on the outcome on the procedure to be followed in case of such emergencies”
	**EBP**	SOPs and protocols are adapted from WHO guidelines to local settings is crucial for neonatal care and integrated for evidence-based decisionmaking into neonatal nursing & other healthcare practices	“its important to provide patients and their families with clear information & involving them in the care process to ensure better outcomes and satisfaction.”	“The concept of evidence-based practice is about using evidence in making decisions to improve patient care. For instance, in maternal health, we use tools like the kartograph to monitor labor and make informed decisions based on established protocols and guidelines”
Intuition & Experience in Nursing	**Use of Intuition**	The interviewees acknowledge that while evidence-based & experience also play a significant role in decisionmaking, especially in high-practices are crucial, intuition pressure or resource-limited situations.	“I would go with the intuition because sometimes it's an emergency. In an emergency situation, you're able to assess a mother or a baby who needs urgent intervention. When you're alone they're relying on you especially if you're a team leader they're relying on you to make decisions”	“At times you require to use intuition… When there is a shortage of staff or when the doctor isn't there. You are able to use intuition to guide & be able to recognize a problem earlier so that you can intervene.”
Challenges in Implementing Evidence-Based Practices	staffing time constraints need for continuous training & education balancing workload with staying updated on the latest research.	Both interviews discuss challenges to implement evidence-based practices effectively.	“Staffing issues is another one… Especially during that time of induction it's low. And that is where I know you find the problems. You need a strong person to make decisions especially as a team leader. There is none because of the shortage of the staffs. So you find it a bit difficult to coordinate care during that time”	“The challenge one of them is availability of the doctors. Staffing issues is another one…. especially the team there is high turnover of staffing in the whole hospital. Let's say the whole hospital” “I didn't like research when I was in school. I see it and I pass it then put it aside, its so complicated” “We have many patients and there's no time to teach so u learn as you continue with work.”
Multidisciplinary Collaboration		Collaboration among different healthcare professionals is a recurring theme. High levels of collaboration between nurses, doctors, and consultants are necessary, though challenges remain due to differing attitudes and behaviors.	“based on protocols from WHO and some we sit with a multidisciplinary team from consultant neonatalists MOs Cos nurses; we come up with SOPs how are conditions meant to be managed?”	“From that angle with the evidence you can be able to coordinate care with other cadres within the departments so that you can improve the outcome.”
Support for Professional Development	Enabling factors Barrier factors	Highlight the support provided by hospitals for the professional development of nurses, including access to education, study leave, and resources like libraries and computers. However, financial constraints and workload come in	“There is a fully-fledged unit within the nursing department which deals with CME continuous medical education and students. So there are schedules like now which is ongoing. There are trainings which are ongoing to improve patient's care if there's a need.”	“Need to strengthen systems and a lot of training like the continuous medical education to be done on a regular basis rather than waiting would improve patient care. The challenges of this is the logistics in planning for workshops education programs … If there is facilitation to do those”
Challenges in doing Research	Complexity Lack of Support Interest Motivation	Research is perceived as complicated and stressful, often disliked by practitioners during their studies, a need for better support in understanding & conducting research, as many feel they have to struggle on their own. The drive for professional development varies among individuals, influenced by personal motivation & the support	“If there is that positive attitude from my employer, it'll drive more nurses to say if I go study more which means I am capacity building myself, I move to this level and given this rank, this work to do then itll create a motivational impression on nurses and the will move” “If one wants to study, we have the library in the school as the school is part of the hospital”	“You will find one who is in the field of nursing recently, few years but has interest. So one has, the other doesn't have interest (in training) so it'll depend on individual and what is driving the individual” “CME room. Okay. It has the infrastructure. That's a department in itself.”
Continuous learning		Continuous training, especially in critical skills like neonatal resuscitation, is necessary for both nurses & doctors with the institution providing infrastructure, opportunities for education, & study leave is crucial for professional growth	“There are trainings which are ongoing to improve patient's care if there's a need. So, basically, I would say there is improvement, there is support from management, because the participants of those CMEs, those training opportunities.”	“If there is that positive attitude from my employer…………. it will create a motivational impression on nurses and the will move”

## DISCUSSIONS

This discussion section brings an integrated approach to discussing the results from both the quantitative and the qualitative data. Due to the complexities of decision-making in nursing practice the overarching aim of the study was to determine the factors related to EBDM & P among specialized nurses. Understanding of nurse decision-making in the maternal/child environment is therefore essential for enhancing patient outcomes. The primary result of the study was predominant use of intuition among specialized nurses being associated with low utilisation of EBDM.

However, the study needs to be viewed as an exploratory study being indicative rather that definitive. In future research, we recommend confirmatory studies with appropriate adjustments such as Bonferroni correction or False Discovery Rate control that would recruit multiple and larger samples.

Results of the study shows that respondents have basic knowledge of the use of evidence in patient care and how to implement them. This implementation being done though SOPs, protocols and guidelines. It is noted that there are some specialized tools used for EBDM EBP that are being adapted from global partners.

However a significant number were neutral in responding to the questionnaire. Reasons for being neutral could be lack of understanding of the subject matter and or ambivalence of the discussion matter, alluding to inexperience of the respondents. This can be seen aligning to the age of the respondents who were majority in the lower age bracket.

### Evidence vs Intuition

Intuition has been considered the “art of nursing” or “aesthetic knowing”, and “tacit knowledge” or “personal knowing”; a “gut feeling”.^15, 16^ Intuition was first described by Hamm as ‘going beyond merely a lack of analysis and including an experienced decision-makers’ depth of knowledge for facilitating an ability to predict circumstances effectively.^17^ According to Hamm, traditional education of medical students involves teaching a systematic approach to decision-making, alluding to use of evidence. However it is seen that experienced decision-makers (including nurses), appear to make decisions without obviously following a formal decision-making procedure; This Hamm called use of Intuition.^17^ In the nursing profession, there are unconscious processes that facilitate their decision-making. These unconscious processes are largely based on experience.^18^ Experience and intuition have been studied as ingredients for nurses’ decision making.^18^ In this study the thought paradigm emerged as a significant issue in utilisation of EBDM a with a *p* value of .039, meaning that it contributes majorly towards the use of EBDM the effect being unlikely to have occurred by chance. Majority; 85.7% of respondents in the study were seen to apply intuition rather than evidence in decision making, this finding emphasizing how clinical intuition contributes to patient-centered care. It reflects the reality that EBDM&P involves applying research findings, while integrating experiential knowledge and professional judgment. It is noted however that some respondents still preferred evidence use and advocated for the same. This shows a combination in the use of the two thought paradigms that begs further probing.

The combination of intuitive and analytical (evidence-based) approaches has been observed in literature as enabling medical decision-makers, with varying levels of experience, to make decisions in a variety of situations with differing contextual features.^18^ This literature seems to support the results of our study.

Use of intuition varies among nurses. According to literature, use of intuition is associated with increased age and experience among nurses.^19^ Intuitive nurse decision-making is based on years of experience and includes, recognition of similarities between patient care situations, awareness developed over time, and a process that may appear to be without rationale. Nurses with more experience prefer using intuition in their practice as Intuition provided a confidence in nursing skills, employs new nursing practice methods, as well as a feeling of connection with patients.^20^ However the case of this study revealed that majority of respondents who preferred intuition in the study were of lower age and years of experience. A similarly contradiction emerged in a study by Parker in 2014, where older age and longer experience was correlated with evidence based decision making among decision makers^21^ unlike our case where shorter experience was correlated with evidence based decision. Qualitatively, respondents agreed on the importance of intuition further going on to correlate use of intuition with staffing constraints.

The use of intuition has been linked to connectedness with patients. In a study conducted by Hassani in 2016, it was noted that nearly all the nurses in that study indicated that their intuition inspired them to re-assess the prognosis of patients, to that of poor conditions; conditions that were previously not detected by typical physical examinations and laboratory findings.^16^ Similarly in this study, nurses felt connected with their patients whereby majority (62.8%) strongly agreed that they were connected with their patients again alluding to the relying on Intuition. As majority of respondents alluded to reliance on Intuition, so did they also strongly agree that the use of intuition boosted their confidence in their patient care. Similarly, Intuitive nurses have been characterized by their confidence in intuition, and willingness to take unconventional approaches to problem-solving^19^, more so in the complex environment of clinical care. Confidence which is developed with time just like experience is vital in the nursing profession for building of trust with patents and their families; trust that the patient is being given utmost care, avoidance of self-doubt and eventually positive patient outcomes^22^. In response to earlier research question ‘What is predominantly used by specialized nurses when making decisions for cancer service delivery; intuition or evidence?’ and objective of ‘Assessing the weight of use of intuition vs evidence thought paradigms in EBDM among nurses in selected facilities in Nairobi’ it emerged that Intuition is the most applied thought paradigm among reproductive nurses working in the level 4, 5 and 6 in Nairobi.

There emerged a contraction between nurses’ decision making thought paradigms; using intuition and the working environment being of low autonomy. This alludes to the concept of bounded autonomy. The environment in which nurses operate, is prevalent with strict protocols, hierarchy decision making and physician led directives showing limited formal autonomy. However nurses can still be seen to exercise a high degree of practical autonomy in real-time clinical situations, when they are under pressure and or in rapidly evolving patient scenarios. In these moments they draw on tacit knowledge developed through experience, pattern recognition, and situational awareness using intuition.

### Institutional Factors

Institutional factors emerged as not significant to EBDM. This means that the effect may not be important, however this is discussed with caution as the small sample size may have contributed to this. According to Nibberlink & Brewer, organizational decision-making factors within a nursing unit provide informal influence over nurse decision-making that could influence patient care.^18^ The indicators studied under Institutional factors were organizational culture, research infrastructure, guidelines and document support and decision making autonomy. It emerged that the ‘hard’ infrastructure is availed, some through a library, some through a facility which is linked to a nursing school and or through set up of specific departments.

However, respondents in majority disagreed on the availability of stable internet connection (31.4%). Research infrastructure here included stable internet provision and computer equipment that is used to access information and knowledge that can guide decision making. With research infrastructure unavailable or limited, access to input for decision making is questionable for reproductive nurses working in the public sector in Nairobi. According to several authors, developing countries are noted to remain technically challenged and financially under-resourced hence having shortfalls on the availability of evidential data and insight required to make informed decisions.^22, 24^ This case in clearly seen in this population. Qualitative results show that the organization factors also include balance of workload which is challenging towards engaging in research for EBDM, once again being correlated with staffing and time constraints.

In terms of autonomy of decision making, respondents were mostly agreeable on the need to have supervisory approval before decision making where guidelines are not available and or not clear (29.4%), although marginally. This indicates the low levels of autonomy for the population in decision making. This practice of ‘consulting’ however contradicts with the results that revealed that majority of the nurses leaned on intuition for decision making. Similarly, autonomy was also studied by Kassie, Tadele and Beza et al where approximately 44.9% of respondents in that study indicated that a lack of autonomy in changing practices was a significant barrier to EBP^25^ with similar conclusions on the positive influence of autonomy on EBDM.

According to authors, decision making among nurses is equated with professionalism. Hence to enhance professionalism, one requires more autonomy indecision making, become a person who thinks, makes decisions, and takes responsibility for his/her decisions, not just someone who takes orders.^26^ This is also correlated with years of experience and subsequently use of intuition. While quantitatively majority of respondents were neutral on the organizations’ culture towards sharing and facilitating EBDM, qualitatively, respondents noted that organizational constrains including work allocations from the facility management limited their interactions with EBDM. This is alluding to culture that doesn't support engagement with EBDM.

### Individual Factors

The study looked at the indicators of individual factors as complexity of research, skills for engaging in research, language of research and application of EBDM. According to authors, most African countries lack access to EBDM resources and well-trained public health personnel who have the capacity to predict, prevent, respond and control epidemiological events before disease outbreaks spread beyond local areas.^27^ Although this is referring to health workers as a whole; it equally applies to nurses also needing these skills for EBDM. In qualitative interviews respondents noted that individual drive is critical to acquiring skills for engaging with research and learning. Individual factors seemed to have the highest significance to the use of EBDM with a *p* value of .001. Responses from questions on individual capacities revealed that they have requisite skills (45.1%) however they do understand the language of research (43.1%); although this is less than half of the participants. Individual factors can then be interpreted that the most affective factor to the utilization of EBDM among this group.

Respondents strongly disagreed that they do have incentives to engage with research (27.5%). What incentivizes nurses in participating in research have been described in literature including: lack of knowledge, training and skill, mentoring support and protected time.^28, 29^ Severally respondents in the study mentioned the limitation of time to engage in research and or evidence due to shortage of staff, including qualitatively.

Nurses in general hospitals perceived low levels of evidence-based practices according to Nwe et al with the indicator attitudes/perceptions (*p* =.001) being significant to EBDM.^29^ Similar to our study, this study unearthed time as a constraint to EBDM among nurses.

In a study by Dalheim et al conducted on nurses and titled ‘Factors influencing the development of evidence-based practice among nurses’, the availability of skills in finding, reviewing and using different sources of evidence were noted as critical to EBDM. These were positively seen as barriers in use of research evidence.^30^

### Barriers to EBDM

Literature tells us that barriers to EBDM include organization support, continuous education systems, improved skills, knowledge, and confidence (alluding to autonomy)^31^. With the research question, What are the barriers to the access of maternal and pediatric research evidence for EBDM by specialized nurses for patient service delivery Cumulatively respondents strongly agreed that there exists barriers in their respective facilities. These included time (Quant 29.4%), staffing constraints (qual), access (Quant 27.5% and 29.4%), and skills (qual).

In the qualitative results, one facility respondents expressed that training and continuous learning was not a barrier, rather an enabler and there was management support for the same. In another facility however, some challenges were seen in this area. This means that opportunities for self-development through continuous learning of the nurses was contextual to a facility. Organizational support for continuous development also emerged as contextual to a facility. It is worth noting that quantitatively, majority of respondents were neutral in acknowledging the availability of general organizational support, (47.1%).

A review of research in this area showed that nurses indicated a preference for information provided by experienced colleagues or their own experience more than other sources of information^32,34^, more so as finding access to colleagues more efficient and patient specific in time constrained critical decision-making circumstances. This showed collaboration and joint decision making (what is called in literature, Shared Decision making) among the reproductive nurses studied. Literature supports the use of shared decision making for EBDM and EBP and recommends the same for enhancing and embracing evidence among nurses^33^. In this study, shared decision making was evidenced when respondents strongly agreed that there is a culture of joint decisions. (37.3%).

Dalheim et al noted insufficient time as a reflection of mental time and energy required to use research and the culture of ‘busyness’ rather than the actual amount of time required. This correlates with institutional factors such as culture being a barrier to EBDM & P. Such a culture of rewarding those who seem to be actively busy, not idle sitting and reading, does not favor participants who reflect over research results for integration into practice^30^. Though this was not explicitly mentioned in this study, it was mentioned that the time for ‘reading’ was limited. Dalheim's study went on to conclude that processes (in nursing) should include courses in evidence-based practice for nurses.^30^ A key barrier that kept emerging in EBDM & P, is time. Barako et al in a study conducted with Kenyan nurses, also notes that major limitations to EBP application includes time^34^. Barriers to access emerged as not significant to EBDM. This is to be again taken with caution as some of the barriers are individual in nature, hence some form of contradiction is seen here.

### Study Limitations

The limitation of this study was that the study findings cannot be generalized to other realms or sectors; the reporting of the results is therefore limited to nurses working specifically in reproductive wings of a public facility in Nairobi. To address this limitation, longitudinal studies will be required to determine causal judgments between evidence based decision making and specialized nursing practice. The number of these nurses is notably low, hence the limitation of the multivariable models used. To delimit this, we recommend that future studies with larger samples be conducted to ensure the findings and allow for more complex modeling which have greater statistical power, including other specializations of nurses and or private sector nurses.

## CONCLUSION & RECOMENDATIONS

The study provided an improved understanding of decision-making among specialized nurses in public sector in this environment (Nairobi) that may help to guide future efforts to support nursing practice for enhanced patient care. As a whole, the knowledge that is mainly relied upon for decision making among reproductive nurses working in the public sector in Nairobi was tacit knowledge. This being exercised through the use of intuition rather that explicit knowledge that is exercised through documented procedures and policies. Further, individual factors such as skill, incentives and understanding of research are critical to enhancing of EBDM among specialized nurses working in public health facilities.

Tacit knowledge which is knowledge domiciled within individuals, is gained through time and experiences that is context-specific and difficult to articulate, formalize, or transfer through traditional communication channels. As it emerged as a key influencer of EBDM & EBP, specific strategies and mechanisms for ‘harvesting’ tacit knowledge are recommended to be developed and implemented in the public health sector more so for specialized nurses. This will enhance transfer/translation of knowledge and facilitate nuanced EBDM.

Building of skills for engagement with research among specialized nurses is a key challenge identified in this study. Introduction of EBDM to nursing curricula early will enhance adapting EBDM&P elements among nurses right from the beginning of their careers while infusing positive perceptions for continuous engagement with evidence.

More nuanced research especially longitudinal studies with larger sample sizes and or multidisciplinary respondents to tease out and further explore factors and their influences with EBDM are suggested.
